# Electrophysiological Potentials Reveal Cortical Mechanisms for Mental Imagery, Mental Simulation, and Grounded (Embodied) Cognition

**DOI:** 10.3389/fpsyg.2012.00329

**Published:** 2012-09-14

**Authors:** Haline E. Schendan, Giorgio Ganis

**Affiliations:** ^1^School of Psychology, University of Plymouth, PlymouthDevon, UK; ^2^Massachusetts General Hospital, Athinoula A. Martinos Center for Biomedical ImagingCharlestown, MA, USA; ^3^Department of Radiology, Harvard Medical SchoolBoston, MA, USA

**Keywords:** mental imagery, visual shape perception, object categorization, face identification, semantic memory priming, visual knowledge, embodiment and grounded cognition, event-related potential

## Abstract

Grounded cognition theory proposes that cognition, including meaning, is grounded in sensorimotor processing. The mechanism for grounding cognition is mental simulation, which is a type of mental imagery that re-enacts modal processing. To reveal top-down, cortical mechanisms for mental simulation of shape, event-related potentials were recorded to face and object pictures preceded by mental imagery. Mental imagery of the identical face or object picture (congruous condition) facilitated not only categorical perception (VPP/N170) but also later visual knowledge [N3(00) complex] and linguistic knowledge (N400) for faces more than objects, and strategic semantic analysis (late positive complex) between 200 and 700 ms. The later effects resembled semantic congruity effects with pictures. Mental imagery also facilitated category decisions, as a P3 peaked earlier for congruous than incongruous (other category) pictures, resembling the case when identical pictures repeat immediately. Thus mental imagery mimics semantic congruity and immediate repetition priming processes with pictures. Perception control results showed the opposite for faces and were in the same direction for objects: Perceptual repetition adapts (and so impairs) processing of perceived faces from categorical perception onward, but primes processing of objects during categorical perception, visual knowledge processes, and strategic semantic analysis. For both imagery and perception, differences between faces and objects support domain-specificity and indicate that cognition is grounded in modal processing. Altogether, this direct neural evidence reveals that top-down processes of mental imagery sustain an imagistic representation that mimics perception well enough to prime subsequent perception and cognition. Findings also suggest that automatic mental simulation of the visual shape of faces and objects operates between 200 and 400 ms, and strategic mental simulation operates between 400 and 700 ms.

## Introduction

Mental imagery is the ability to reactivate and manipulate modality-specific mental representations when current sensory stimulation or overt motor action is absent, and this ability can be associated with the subjective experience of perceiving or acting within one’s mental world (e.g., “seeing with the mind’s eye”). Mechanisms of mental imagery have been proposed in large scale network theories (Kosslyn et al., 2006). At an abstract level, such imagistic theories propose that, during mental imagery, modality-specific, long-term memory is reactivated in a top-down manner and maintained via working memory processes so that they can be inspected and manipulated to achieve a task goal (Kosslyn, 1994; Kosslyn et al., 2001; Ganis et al., 2003; Ganis and Schendan, 2011). Notably, the mechanisms proposed in these theories of mental imagery resemble those in grounded (embodied) cognition theory, which proposes that cognition is grounded in modal processing of sensorimotor information and introspective states (e.g., emotion, motivation, intention; Pulvermuller, 1999; Barsalou, 2008). Like imagistic theories of mental imagery, grounded cognition theory challenges the dominant symbol systems paradigm inspired by formal theories of logic, language, and computation that proposes that amodal symbol representations, which are independent from the sensorimotor processes, support language, thinking, attention, memory, and meaning (Fodor, 1983; Johnson-Laird, 1983; Pylyshyn, 2003). Recently, however, tests of grounded cognition theory have yielded compelling evidence that modal processing affects cognition, including meaning, even when task irrelevant, and *vice versa* (e.g., Tucker and Ellis, 1998; Wilson, 2002; Vigliocco et al., 2006; Barsalou, 2008; Fischer and Zwaan, 2008; Kemmerer et al., 2008; Chatterjee, 2010; Anderson et al., 2011; Hirschfeld and Zwitserlood, 2011; Meteyard et al., 2011). However, little is yet known about when, how, and how much cognition is grounded and about the brain mechanisms (Mahon and Caramazza, 2009; Chatterjee, 2010; Rumiati et al., 2010; Meteyard et al., 2011). The main proposal for how cognition is grounded is mental simulation, defined as the “re-enactment of perceptual, motor, and introspective states acquired during experience with the world, body, and mind” (Barsalou, 2008, 2009). The present experiment aims to reveal the cortical dynamics of mental imagery mechanisms that may ground cognition in mental simulations. Crucially, the cortical dynamics of mental imagery and mental simulations that ground cognition are almost entirely unknown because electromagnetic brain sensing methods, which reveal neural activity directly with the required high time precision [milliseconds (ms)], have not been applied, as done here.

An important distinction in grounded cognition theory to consider is that between non-conscious automatic simulations implicated, for example, in constructing meaning from language (Bub and Masson, 2010; Wassenburg and Zwaan, 2010) and conscious effortful simulations, such as mental imagery, that result from conscious representations constructed in working memory strategically (Kosslyn et al., 2006; Barsalou, 2008, 2009). Critically for the present study, both types of simulation share common representations (Barsalou, 2008; Moulton and Kosslyn, 2009). We hypothesize that, at the level of brain mechanisms, the top-down feedback mechanisms that support automatic simulation are a subset of those that support mental imagery (and conscious effortful simulations; Ganis and Schendan, 2011). Specifically, we propose that non-conscious automatic simulations unfold via reflexive top-down signals from higher to lower level areas along modal information processing pathways (e.g., the ventral stream for processing visual objects): Perceiving a stimulus triggers these processes reflexively. These same processes are triggered by effortful, strategic, task-oriented, top-down signals from the prefrontal cortex during mental imagery (Ganis and Kosslyn, 2007), which also triggers them most strongly due to the deliberate, targeted nature of the task requirements. Thus, studying mental imagery provides a powerful way to reveal the maximum set of top-down feedback mechanisms that support mental simulation, including non-conscious automatic simulation.

To ensure that mental imagery mechanisms underlie the effects, this experiment used a validated mental imagery task (Kosslyn et al., 2006). The key task elements are that subjects first memorize pictures of faces of real people or objects extensively and then practice visualizing these pictures mentally. Afterward, during the mental imagery task, the name of the person (or object) cues mental visualization of the associated picture of the face (or object). The critical and novel element of this design is that, after subjects report (by pressing a key) that they have generated a vivid mental image, a target picture appears 200 ms later. This picture is either identical to the picture that they had learned to visualize (*congruous condition*) or different from it, being from the opposite category (i.e., if a face was visualized, an object is shown, and *vice versa: incongruous condition*). The impact of the imagined picture on the target picture is used to define the cortical dynamics of mental imagery. The advantage to comparing mental imagery of these two categories is that faces and objects recruit different posterior visual processing areas (Hasson et al., 2003; Downing et al., 2006) and are associated with distinct ERP signatures (e.g., Schendan et al., 1998; Allison et al., 1999; Puce et al., 1999). This experiment thus reveals when top-down processes for mental imagery of visual shape can ground cognition of faces and other objects. Such neurophysiological markers will be crucial for future work on when, how, and how much top-down cortical processes of mental simulation ground cognition.

Findings from this experiment have been reported already for early ERPs before 200 ms (Ganis and Schendan, 2008), that is, the vertex positive potential and its occipitotemporal N170 counterpart (VPP/N170), which are associated with categorical perception. The goal here was to analyze the later ERPs that were not analyzed previously and are associated with knowledge, meaning, and category decisions. These abilities are the main targets for grounded cognition explanations. The main hypothesis is that, if mental simulation constructs cognition, including meaning, then mental simulation of modal processes induced by mental imagery (and the associated cortical dynamics revealed by the ERPs) should resemble those associated with cognitive and semantic effects. If true, then this would constitute crucial evidence that links mental simulation of modal processing (using top-down mental imagery mechanisms) with cognition and meaning, as proposed in grounded cognition theory. To assess cognitive effects, this study capitalizes on ERP markers of face and object cognition (Neumann and Schweinberger, 2008; Schendan and Maher, 2009). To assess semantic effects, in particular, this study capitalizes also on well-studied semantic congruity and priming effects. These effects are thought to reflect processing meaning in the semantic memory system (Rossell et al., 2003) and to operate via the same automatic top-down processes implicated in automatic mental simulation (Collins and Loftus, 1975; Franklin et al., 2007; Kutas and Federmeier, 2011).

To predict the specific pattern of ERP cognitive, semantic congruity, and priming effects that mental imagery could produce, this report capitalizes on the two-state interactive (2-SI) account of the brain basis of visual object cognition (Schendan and Kutas, 2003, 2007; Schendan and Maher, 2009) and extends it into a multi-state interactive (MUSI) account. This framework proposes that posterior object processing areas activate at multiple times in brain “states” serving distinct functions. State 1: Initial activity in object processing areas feeds forward from occipital to temporal cortex between ∼120 and ∼200 ms when a visual object is coarsely perceptually categorized, indexed by the VPP/N170 (Schendan et al., 1998; Schendan and Lucia, 2009). State 2: Object processing areas activate again but in a sustained, interactive manner dominated by feedback and recurrent processing among these areas and ventrolateral prefrontal cortex (VLPFC) between ∼200 and 500 ms, indexed by a frontal N3(00) complex (often labeled frontal N400). The N3 is specific to processing pictures of a face or object and non-linguistic information (e.g., shape; Barrett and Rugg, 1989, 1990; McPherson and Holcomb, 1999; Nessler et al., 2005) and is the first ERP to modulate according to visual cognitive factors that similarly affect object processing areas and VLPFC, including semantic memory (Barrett and Rugg, 1990; Zhang et al., 1995; Doniger et al., 2000, 2001; Schendan and Kutas, 2002, 2003, 2007; Philiastides and Sajda, 2006, 2007; Philiastides et al., 2006; Schendan and Lucia, 2009, 2010; Schendan and Maher, 2009). For example, the N3 becomes more negative with greater mental rotation (Schendan and Lucia, 2009), worse speed and accuracy of category decisions, greater stimulus atypicality and impoverishment (e.g., atypical views, visual degradation; Doniger et al., 2000; Schendan and Kutas, 2002, 2003; Johnson and Olshausen, 2003), and implicit memory (repetition priming, i.e., better decisions for repeated than new objects; Henson et al., 2004; Schendan and Kutas, 2007) primarily for meaningful objects (e.g., dog; Voss et al., 2010). Such effects also localize to object processing areas (David et al., 2005, 2006; Philiastides and Sajda, 2006, 2007; Sehatpour et al., 2008; Schendan and Maher, 2009; Schendan and Lucia, 2010; Clarke et al., 2011). Later in state 2, the well-studied, centroparietal N400 between 300 and 500 ms reflects interactive activation of semantic memory in anterior temporal cortex and VLPFC, especially in response to words (i.e., linguistic stimuli; Marinkovic et al., 2003; Lau et al., 2008; Kutas and Federmeier, 2011). Both mid-latency negativities (N3 and N400) are more negative for stimuli that are incongruous (i.e., non-matching) relative to congruous (i.e., matching) with the immediately preceding semantic context based on a sentence or scene (Ganis et al., 1996). A similar account has been proposed for face cognition (Neumann and Schweinberger, 2008; Burton et al., 2011).

The MUSI account revises this story by adding State 3 that operates after about 400 to 500 ms and performs internal evaluation and verification processes, including conscious effortful mental simulations. For example, later verification of category decisions, more complex semantic processes, and episodic memory have been associated with a posterior late positive complex (LPC) after ∼500 ms (Schendan and Kutas, 2002; Rugg and Curran, 2007; Voss et al., 2010) that is, instead, more positive to incongruous than congruous semantic contexts with objects, videos, and faces (Ganis et al., 1996; Schendan and Kutas, 2002; Ganis and Kutas, 2003; Sitnikova et al., 2009, 2010). Altogether, this predicts that the N3 and N400 will be more negative and the LPC will be more positive when mental imagery does not match the current picture (incongruous) than when it does match (congruous). Finally, cortical sources of the ERPs to faces and objects and associated congruity effects should differ because these two categories recruit distinct cortical areas (Hasson et al., 2003).

Prior work on mental imagery and semantic congruity and priming would not necessarily predict such later effects, however. The only prior ERP study with a similar mental imagery task revealed no effects after 300 ms, predicting no effects here (Farah et al., 1988), but that study involved imagery of two letters, which have minimal meaning. In contrast, the present work aims to reveal mental imagery for stimuli with richer semantic content. Studies of semantic congruity and priming effects would also predict no ERP effects of mental imagery after 200 or 300 ms because the slow stimulus timing and cross modal conditions here (i.e., word cue followed by picture) violate standard methods for producing such semantic effects, as detailed in the discussion. To anticipate, results reveal effects not only on the early VPP/N170 during categorical perception, as reported previously (Ganis and Schendan, 2008), but also later ERPs (N3, N400, and LPC) linked to knowledge, meaning, and categorization, as well as a P3(00) linked to immediate repetition priming of perceived faces and objects (Bentin and McCarthy, 1994; Nielsen-Bohlman and Knight, 1994). In addition, we report results of new analyses of data from the perception control experiment conducted in the same group of participants (Ganis and Schendan, 2008) that had not been explored previously (i.e., face ERPs after 500 ms and all object ERPs). As ERPs to faces starting from the early VPP/N170 until 500 ms show perceptual adaptation (i.e., reduced for congruous; Ganis and Schendan, 2008), the N3, N400, and LPC ERPs should likewise show adaptation.

## Materials and Methods

**Figure 1:**
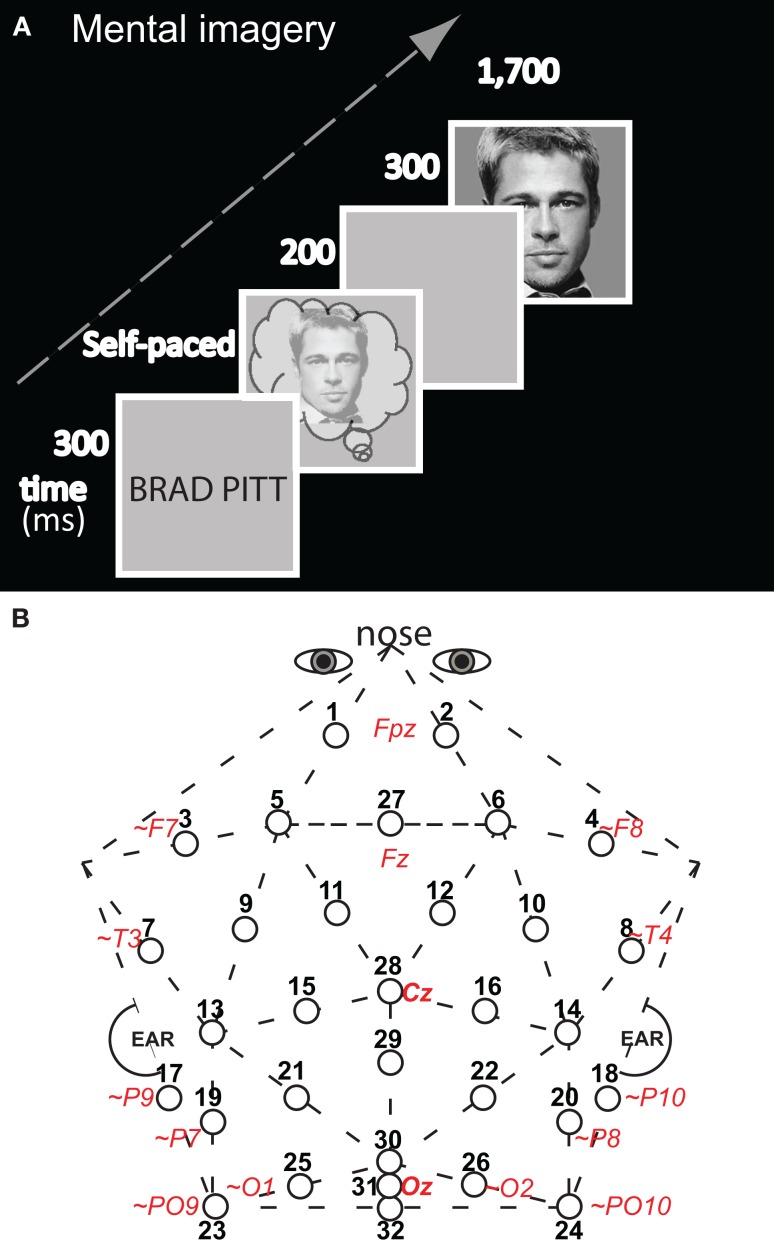
**Methods**. **(A)** Diagram of an experimental trial for the test of mental imagery for faces and objects (only a congruous trial of face imagery – face picture is shown). An appropriate face (or object, not shown) was visualized mentally upon seeing the corresponding name (which was on the screen for 300 ms). Subjects pressed a key as soon as they had generated a vivid mental image, and 200 ms after this key press, the test picture appeared, which was either the identical picture that had been imaged mentally (congruous condition) or not (in which case the picture was from the other category: incongruous condition). There was no task on the test pictures. Perception trials (not shown) had a parallel structure to imagery, except that the trial started with perception of a picture of a face (object) was presented until subjects pressed a key as soon as they identified it. **(B)** Thirty-two-channel geodesic montage for EEG recording. Circles show each electrode location with its numerical label. Actual or approximate (∼) locations of 10–10 sites are italicized and in red; posterior sites 23, 24, and 32 lie 1 cm below the inion.

Participants, materials, design, procedures, and electroencephalographic (EEG) recording methods for these mental imagery and perception control experiments were detailed previously (Ganis and Schendan, 2008). This section summarizes key aspects of the methods for understanding this report. The mental imagery experiment started with memorization of pictures of 11 faces of real people and 11 common real objects across 13 exposures each. Next, participants practiced mentally visualizing each memorized picture three times. For this imagery practice, the name appeared followed by a gray screen during which subjects visualized the picture of the named face (object). Once they had done so, they pressed a key to see the actual picture in order to adjust their mental image. For the mental imagery test (Figure 1A), the name appeared for 300 ms followed by a gray screen during which subjects visualized mentally the associated memorized picture. As soon as they had generated this mental image, they pressed a key. Two-hundred milliseconds after the key press the test picture appeared for 300 ms. The test picture was either the picture of the face (object) that was visualized mentally or a picture from the other category [i.e., an object (a face)]. The two by two design included within-subject factors of image congruity (congruous, incongruous) by category type of target picture (face, object). There were 55 trials for each of the four critical combinations (image-picture target pairings) of congruous (face–face; object–object) and incongruous (object-face; face object) conditions. Stimuli were presented on a 21″ cathode-ray tube monitor (1,024 × 768, 150 Hz refresh, Dell P1130) using custom-made *StimPres2.0* software for the *Neurocog* system that ensures precise stimulus time-locking to the EEG (Holcomb, 2003). EEG was recorded at 250 Hz (bandpass .01–100 Hz) from 32 Ag/AgCl electrodes attached to a plastic cap (Figure 1B) and electrodes attached via adhesive to the nose, right and left mastoids, underneath the right eye, and lateral to each eye. The perception control experiment with the same subjects was identical, except for the following. There were no memorization and imagery practice sessions, and participants were shown a picture instead of a name before the test picture: The two pictures were shown consecutively with a 200 ms interstimulus interval (ISI), and the first picture was shown until the participant pressed a key to report that they had identified the object (or face). There were 66 trials in each of the four critical conditions. The primary goal of the control experiment was to replicate perceptual adaptation of the early VPP/N170, and so, in order to focus on higher-level face (object) processing in posterior category specific cortex, instead of low-level simple features, the first and second pictures were never identical, even in face–face and object–object (congruous) trials (Ganis and Schendan, 2008).

### ERP analyses

ERPs were calculated by averaging EEG to each condition, excluding trials with above threshold muscle activity, blinks, eye, and other movement artifacts, time-locking to test picture onset with a 100 ms pre-stimulus baseline. For analyses, ERPs were re-referenced to the mean of both mastoids, and, for visual comparison with prior work, also to the average of all electrodes, except bilateral eyes. Maps of voltage distribution across the head were produced using *EEGLab* software. Eighteen subjects were analyzed; note, due to a scripting error, 1 of the original 19 subjects was not analyzed, but performance results and visual inspection of ERP patterns showed that all results remained the same with and without this subject.

Data were submitted to analyses of variance (ANOVAs) with within-subject factors of congruity and category type (type). For ERPs, ANOVAs assessed separately the lateral pairs (1–26) and midline sites (27–32). Lateral ANOVAs included within-subject factors of Electrode (13 levels) and Hemisphere (left, right). Midline ANOVAs included within-subject factors of Electrode (3 levels) and Site [frontocentral (27–29), occipitoparietal (30–32)]. If Mauchley’s test indicated violations of sphericity, the Greenhouse–Geisser correction was applied to the *p*-value. For brevity, only critical Congruity and Type effects are reported, as scalp location effects alone are not of theoretical interest.

Mean ERP amplitudes were measured within time periods after 200 ms chosen based on prior studies. (1) As N3(00) complex components can vary in functional modulation in ∼100 ms time periods between 200 and 500 ms (Schendan and Maher, 2009), analyses assessed separately (a) 200–299 ms (frontopolar P250 and related polarity inverted occipitotemporal Ncl/N250; e.g., Doniger et al., 2000; Federmeier and Kutas, 2002; Sehatpour et al., 2006; Schendan and Lucia, 2010), (b) 300–399 ms (frontal N350; e.g., Schendan and Kutas, 2002, 2003, 2007), and (c) 400–499 ms (frontopolar N450 and frontocentral N390; e.g., Barrett and Rugg, 1989; Ganis and Kutas, 2003; Schendan and Maher, 2009). The latter two times (300–500 ms) also included the N400 (Ganis et al., 1996). (2) The LPC was assessed from 500 to 699 ms (Heil, 2002; Schendan and Lucia, 2009). (3) Continuation of effects was assessed from 700 to 899 ms (Schendan and Maher, 2009).

To isolate effects, focal spatiotemporal analyses were run on sites and times for which the face (object) cognition-, congruity-, or priming-related ERP was maximal and overlapped least with others; these location choices were confirmed and adjusted by visual inspection. (1) From 200 to 299, 300 to 399, and 400 to 499 ms, respectively, (a) pair 1-2 assessed the frontopolar P250, N350, and N450, (b) pair 11-12 assessed the frontocentral N350 and N390, (c) and pair 17-18 assessed their polarity inverted, occipitotemporal counterparts (Scott et al., 2006; Schendan and Maher, 2009). (2) These times (200–499 ms) were also assessed at pair 19-20 for the centroparietal N400 (Ganis et al., 1996); note, 200–299 ms was included as visual inspection suggested an N400 onset before its typical 300 ms start. (3) Centroparietal pair 19-20 also assessed the LPC from 500 to 699 ms. (4) Visual inspection indicated that a P3 peaked earlier for congruous (∼375 ms) than incongruous stimuli (∼500 ms). Consequently, early on, the congruity effect is more positive for congruous than incongruous from 300 to 400 ms, and later, from 400 to 700 ms, the effect is in the opposite direction. To capture this, local positive peak latency (i.e., highest peak within ±20 ms to avoid spurious peaks due to high frequency noise) between 300 and 699 ms was assessed in each condition at midline occipitoparietal site 30, based on the location of similar immediate repetition effects on the P3 (Bentin and McCarthy, 1994). P3 mean amplitude was also assessed from 300 to 399, 400 to 499, and 500 to 699 ms at site 30. (5) Since visual inspection suggested frontal effects continued after 500 ms, frontopolar and frontocentral focal analyses were also run from 500 to 699 and 700 to 899 ms. For focal analyses, which are more precise (albeit less comprehensive) than omnibus analyses, congruity by category interactions were assessed further using planned simple effect tests of congruity for each category condition.

For perception control ERPs, analyses were the same as for imagery, except for the following. Face ERP analyses through 500 ms were already carried out and reported (Ganis and Schendan, 2008) and so not duplicated here. ERPs for faces after 500 ms and those for objects after 200 ms were analyzed separately, and, for brevity in reporting these control data, comparisons between faces and objects are reported only for focal spatiotemporal analyses, and omnibus analyses are not reported, but they confirmed the focal results.

### Source estimation

Source estimation methods evaluated whether distinct sources underlie congruity effects between categories and category differences. The inverse problem of localizing the cortical sources of electromagnetic data recorded from the scalp has no unique solution without additional constraints. Standardized low resolution brain electromagnetic tomography (s*LORETA*) estimates the sources (Pascual-Marqui, 2002) by making a maximum smoothness assumption to compute the three-dimensional (3D) distribution of current density using a standardized, discrete, 3D distributed, linear, minimum norm inverse solution. Localization is data-driven, unbiased (even with noisy data), and exact but has low spatial precision due to smoothing assumptions resulting in highly correlated adjacent cortical volume units. A realistic head model constrains the solution anatomically using the structure of cortical gray matter from the Montreal Neurological Institute (MNI) average of 152 human brains as determined using a probabilistic Talairach atlas.

s*LORETA* software computed the sources of the grand average ERP difference waves using data from all sites, except nose and eyes (Pascual-Marqui, 2002). ERP difference data are analogous to the signal changes between fMRI conditions, and, thus, limit the sources to those that could reflect fMRI differences, and difference waves can reveal weaker sources better (Luck, 2005). Data were analyzed with bandpass filter of 0.01–20 Hz, based on the validated sLORETA analyses reported previously for the VPP/N170 (Ganis and Schendan, 2008). Electrode coordinates were digitized using an infrared digitization system, and imported into *LORETA-Key* software. This coordinate file was converted using sLORETA electrode coordinate conversion tools. The transformation matrix was calculated with a regularization parameter (smoothness) corresponding to the signal-to-noise (SNR) ratio estimated for each difference wave separately at each 100 ms time period of interest from 200 to 900 ms relative to the 100 ms baseline.

## Results

These results cover all times and comparisons not analyzed for our prior report on this study, which focused mostly on face ERPs before 200 ms during imagery and perception (Ganis and Schendan, 2008).

### Mental imagery ERPs

**Figure 2:**
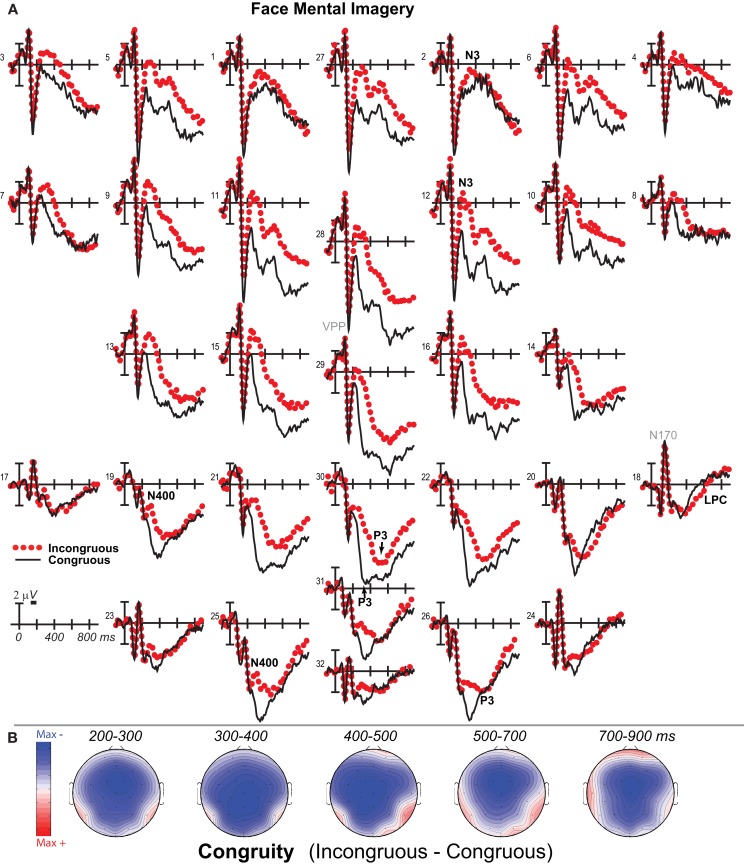
**Mental imagery congruity effects for faces**. **(A)** Grand average ERPs between −100 and 900 ms at all 32 channels shown filtered low-pass 30 Hz. **(B)** Maps of voltage across the scalp for difference waves of the ERPs in **(A)**. VPP, vertex positive potential; LPC, late positive complex.

**Figure 3:**
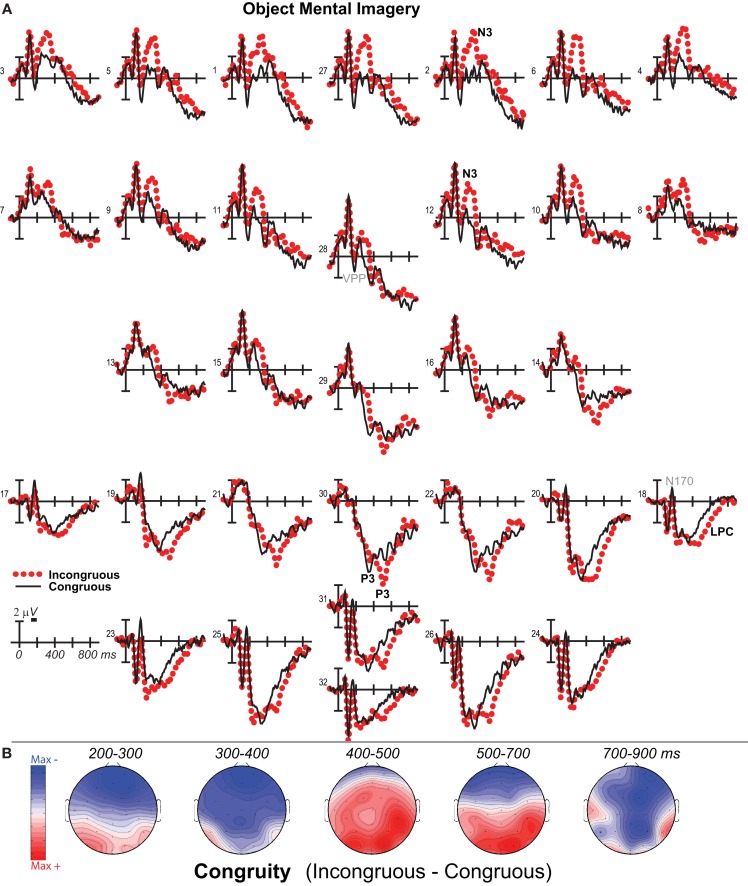
**Mental imagery congruity effects for objects**. **(A)** Grand average ERPs between −100 and 900 ms at all 32 channels shown filtered low-pass 30 Hz. **(B)** Maps of voltage across the scalp for difference waves of the ERPs in **(A)** within each analysis time period.

**Figure 4:**
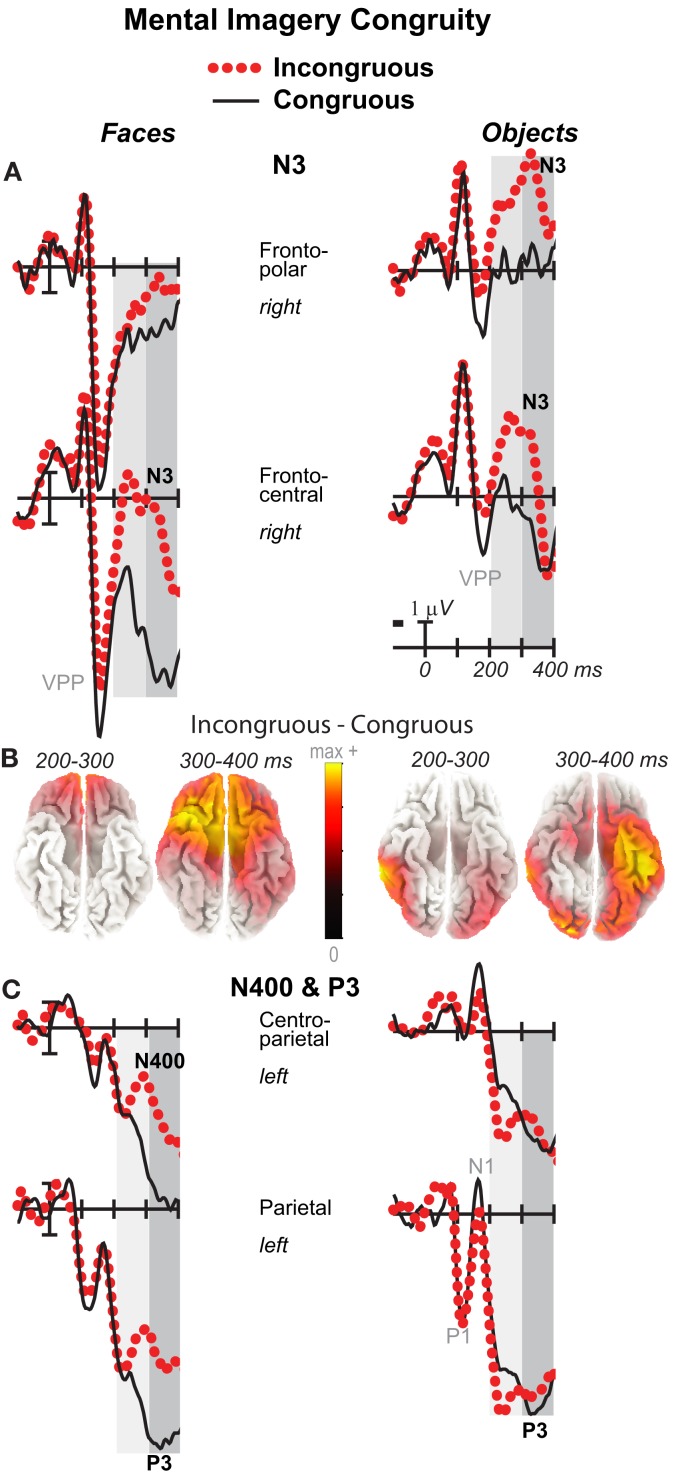
**Mental imagery congruity effects on the N3 and N400 and P3**. Grand average ERPs between −100 and 400 ms at **(A)** right frontopolar (2) and right frontocentral (12) N3 sites and **(C)** left centroparietal N400 (19) and parietal P3 (25) sites. Filtered low-pass 30 Hz. VPP, vertex positive potential. **(B)** sLORETA sources of congruity difference waves at times of the N3, N400, and P3. Images plot the magnitude of the estimated current density based on the standardized electrical activity in each of 6,239 voxels of 5 mm^3^ size.

**Figure 5:**
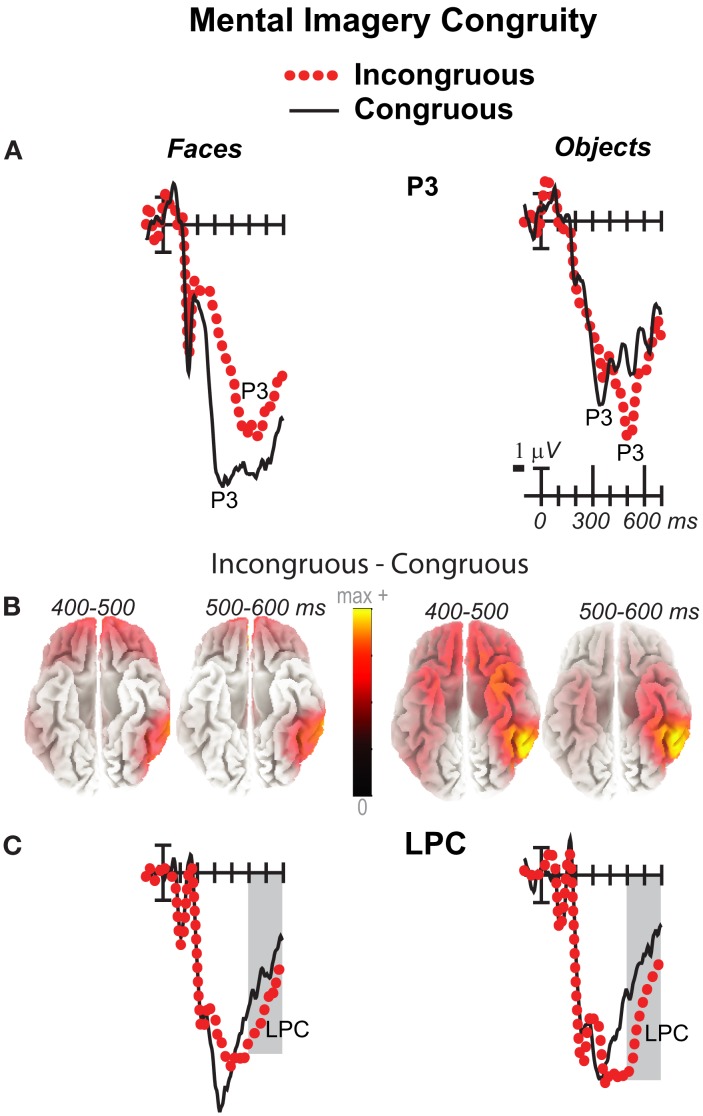
**Mental imagery congruity effects on the P3 and late positive complex (LPC)**. Grand average ERPs between −100 and 700 ms show congruity effects **(A)** on a P3 at a midline parietal site (30) and **(C)** on a late positive complex (LPC) at a right centroparietal site (20). Filtered low-pass 30 Hz. Shading captures the 500–700 ms time when the LPC is maximal. **(B)** sLORETA sources of congruity difference waves at times of the P3 and LPC. Images plot the magnitude of the estimated current density based on the standardized electrical activity in each of 6,239 voxels of 5 mm^3^ size.

**Figure 6:**
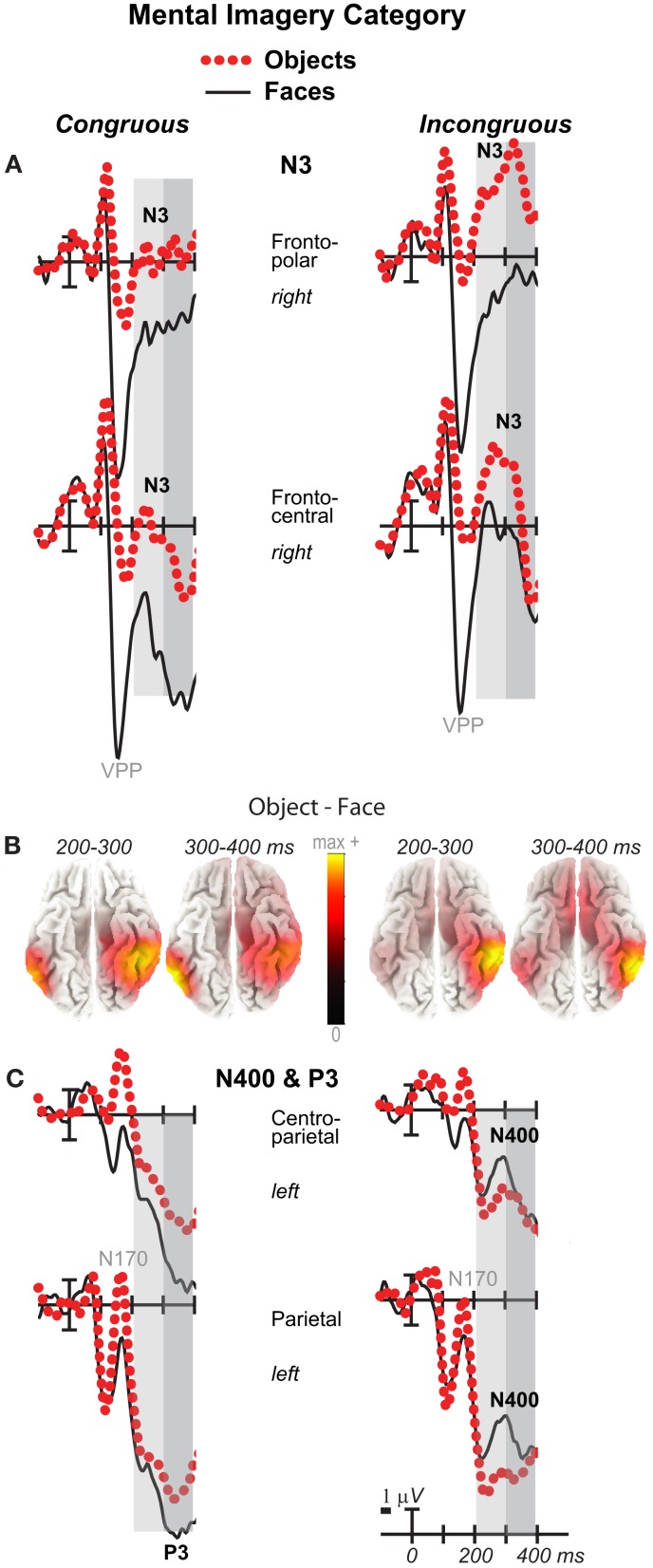
**Mental imagery category effects on the N3 and N400**. Grand average ERPs between −100 and 400 ms at **(A)** right frontopolar (2) and right frontocentral (12) N3 sites and **(C)** left centroparietal N400 (19) and parietal P3 (25) sites. Filtered low-pass 30 Hz. VPP, vertex positive potential. **(B)** sLORETA sources of category difference waves at times of the N3, N400, and P3. Images plot the magnitude of the estimated current density based on the standardized electrical activity in each of 6,239 voxels of 5 mm^3^ size.

**Figure 7:**
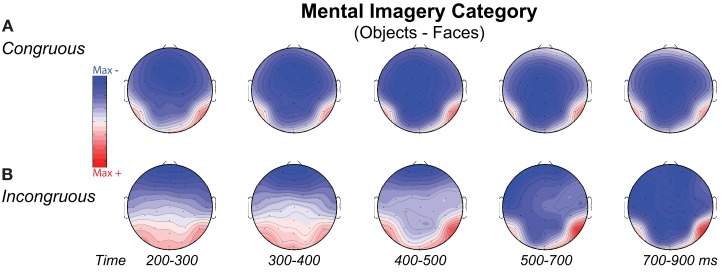
**Volt maps of mental imagery category effects**. Maps plot voltage interpolated across electrode locations in each analysis time window for difference waves that define the category effect on **(A)** Congruous and **(B)** Incongruous trials, as highlighted in Figure 6 for the N3, N400, and P3. Maps produced using *EEGLab* software.

**Figure 8:**
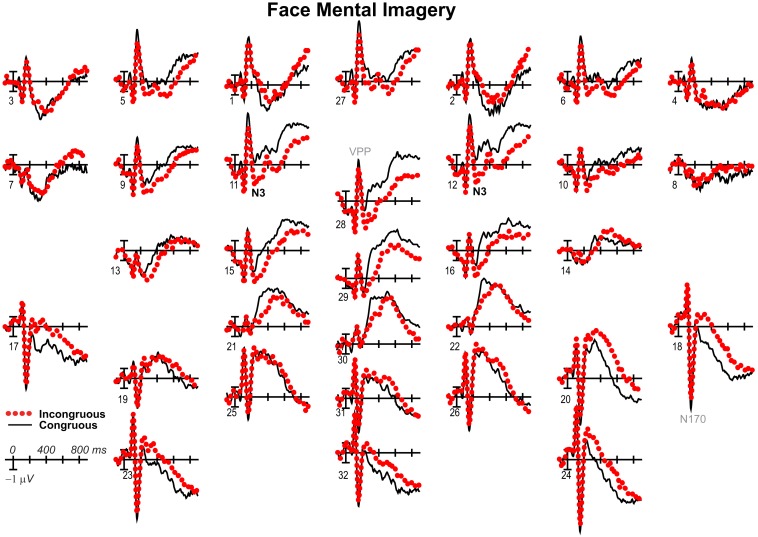
**Mental imagery congruity effects for faces with common average reference**. Same as Figure 2A, except ERPs were re-referenced to the common average and plotted positive up for comparison with some other work.

**Figure 9:**
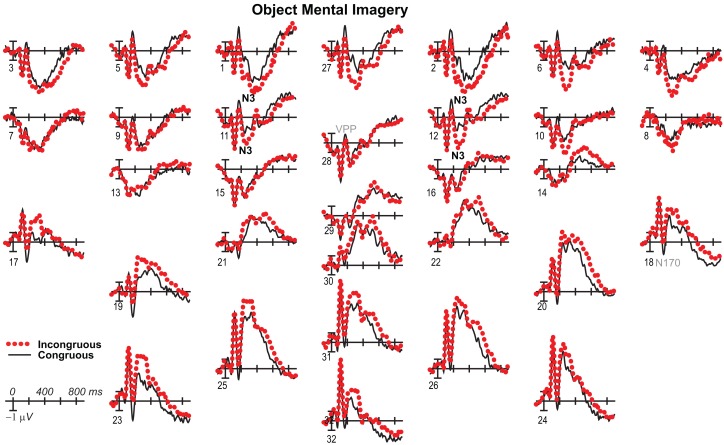
**Mental imagery congruity effects for objects with common average reference**. Same as Figure 3A, except ERPs were re-referenced to the common average and plotted positive up for comparison with some other work.

Mental imagery results for faces during the first 500 ms and objects during the first 200 ms were reported previously (Ganis and Schendan, 2008). The new ERP results here reveal congruity effects to faces after 500 ms (Figure 2), those to objects after 200 ms (Figure 3), and category type effects (Figures 6 and 7) and interactions of congruity by type after 200 ms (Figures 4–6). For comparison with previous work, ERPs are also plotted with the common average reference (Figures 8 and 9). For brevity, (a) only congruity and category type effects, which are of theoretical interest, are reported, (b) degrees of freedom (*df*) are listed only for the first report of each effect, and (c) planned contrasts for omnibus results are not reported, except to note that they supported the corresponding focal spatiotemporal results. For the focal results [all *df*s (1, 13)], any interactions of congruity and category type were followed with corresponding contrast ANOVAs that assessed the congruity effects to objects and faces, separately.

### 200–500 ms: N3, N400, and P3

Negativity on the N3 was greater for incongruous than congruous imagery, and the N400 showed this pattern only for faces (Figures 2–4). Negativity was greater for objects than faces for the N3, regardless of congruity, and the N400 showed this pattern for congruous imagery but showed the opposite for incongruous imagery (Figure 6), consistent with the N400 congruity effect for faces but not objects. Congruity effects had a frontocentral maximum for faces (Figure 2B) and a frontopolar maximum instead for objects (Figure 3B), and, accordingly, object and face categories differed mainly frontally (Figure 7). All effects inverted polarity over occipitotemporal sites. Accordingly, omnibus results (Table 1) showed congruity and category type effects, and interactions of congruity by category type from 200 to 400 ms in lateral and midline ANOVAs and also from 400 to 500 ms in lateral ANOVAs, but showed only category type and congruity by category type interactions in midline ANOVAs from 400 to 500 ms when N3 congruity effects to objects ended.

**Table 1:** ***F*-values for significant effects in lateral (lat) and midline (mid) omnibus ANOVAs with congruity (C) and category type (T) factors at each time period**.

ERP	N3		N3, N400, P3				LPC			
Time (ms)	200–300		300–400		400–500		500–700		700–900	
Source	Lat	Mid	Lat	Mid	Lat	Mid	Lat	Mid	Lat	Mid
Type	14.52**	5.78*	13.03**	8.84**	13.71**	13.26**	20.26**	27.61**	32.67**	32.8**
T × E	23.57**	20.59**	11.91**	11.6**	9.23**	7.78*	7.62**	6.19*	7.5**	8.24*
T × H	–	43.05**	–	20.17**	28.32**	13.15**	49.96**	5.49*	9.53**	8.04**
T × E × H	2.63*	–	–	–	–	6.04*	2.53*	12.56**	2.57*	10.1**
Congruity	12.99**	11.98**	28.72**	26.52**	–	–	–	–	–	5.94*
C × E	18.29**	24.73**	13.52**	14.7**	3.62*	–	9.38**	17.72**	–	–
C × H	–	20.38**	–	6.83**	–	–	–	8.84**	–	–
C × E × H	–	–	–	10.4**	–	–	–	–	–	–
T × C	–	11.62**	8.05*	16.65**	23.51**	23.72**	8.18*	14.38**	–	–
T × C × E	10.16**	–	9.66**	–	6.05**	–	6.89**	–	–	–
T × C × E × H	–	15.93**	–	19.21**	–	7.72**	–	10.93**	–	–

**p < 0.01, *p ≤ 0.05, –*p* > 0.05. E, electrode; H, hemisphere for lateral or anterior-posterior site for midline.

#### N3 complex

Focal spatiotemporal results (Table 2) confirmed frontopolar N3 congruity effects for objects, frontocentral N3 congruity effects for both categories, and centroparietal N400 congruity effects for faces, and occipitotemporal polarity inversion of congruity effects for objects, as well as category type effects. Specifically, results showed type effects on the entire N3 complex from 200 to 500 ms, as frontal negativity and occipitotemporal positivity were greater for objects than faces (Figure 7). Congruity was significant at frontopolar and frontocentral sites from 200 to 400 ms, and, at occipitotemporal sites, congruity was marginal from 200 to 300 ms [*F*(1, 17) = 3.98, *p* = 0.062]; note, while occipitotemporal congruity was also significant from 400 to 500 ms, this was due to the start of the posterior LPC. Congruity interacted with type from 200 to 300 ms at frontopolar sites and from 200 to 500 ms at frontocentral sites (Figures 2 and 3). Planned simple effects tests of the congruity effect for each type showed that this was because congruity effects were largest for objects at frontopolar sites and largest for faces at frontocentral sites and inverted polarity occipitotemporally for objects. Specifically, Table 2 shows congruity was significant at frontopolar sites for objects from 200 to 300 ms and for both category types from 300 to 400 ms, and at frontocentral sites for both from 200 to 400 ms and then later only for faces from 400 to 500 ms. At occipitotemporal sites, congruity was significant only for objects from 200 to 300 ms during the N3 and later from 400 to 500 ms when LPC effects start.

**Table 2:** ***F*-values for significant effects of congruity (C) and category type (T) at lateral electrode pairs and time periods in focal ANOVAs (upper) and corresponding planned simple effects tests of congruity for each category type (lower)**.

Start time (ms)	200	300	400	500	700
**FRONTOPOLAR (PAIR 1–2) FOCAL ANOVA**					
T	45.13**	15.22**	13.29**	5.80*	5.76*
C	11.80**	16.96**	–	–	–
T × C	5.20*	–	–	–	–
*Congruity effect for each category*					
Objects	17.38**	12.28**	–	–	–
Faces	–	5.34*	–	–	–
**FRONTOCENTRAL (PAIR 11–12) FOCAL ANOVA**					
T	17.37**	13.84**	10.56**	18.08**	29.59**
C	23.87**	26.35**	–	9.80**	7.41*
T × C	5.31*	13.60**	21.49**	12.26**	–
*Congruity effect for each category*					
Objects	15.47**	7.94*	–	–	–
Faces	19.96**	31.84**	9.13**	15.25**	9.17**
**OCCIPITOTEMPORAL (PAIR 17–18) FOCAL ANOVA**					
T	9.10**	9.37**	10.79**	–	–
T × H	–	–	7.36*	10.13**	4.85*
C	–	–	4.97*	–	–
*Congruity effect for each category*					
Objects	5.22*	–	7.05*	7.76*	–
**CENTROPARIETAL (PAIR 19–20) FOCAL ANOVA**					
T	–	–	–	8.19*	7.33*
T × H	–	–	23.27**	33.56**	30.06**
C	–	10.42**	5.34*	6.32*	–
T × C	12.27**	5.91*	9.51**	–	–
T × C × H	–	4.63*	–	–	–
*Congruity effect for each category*					
Objects	9.36**	–	23.50**	13.82**	–
Faces	–	12.30**	–	–	–

**p < 0.01. *p ≤ 0.05. –*p* > 0.05. H, hemisphere. Results for 100 ms time periods from 200 to 500 ms; results for 200 ms time periods after 500 ms.

These results and visual inspection indicated that, while frontopolar N3 congruity effects for objects ended by 400 ms, frontocentral congruity effects to faces, as well as type effects at these sites, continued after 500 ms. To assess this, focal analyses of these frontal sites were run on later times. Results (Table 2) at frontopolar sites confirmed no congruity effects there after 400 ms and type effects ongoing between 200 and 900 ms. Frontocentral results confirmed that type effects continued, and congruity effects remained larger for faces than objects until 900 ms, and, indeed, were significant only for faces from 400 to 900 ms. In sum, frontopolar and occipitotemporal object congruity effects occurred between 200 and 400 ms, whereas frontocentral face congruity effects occurred from 200 to 900 ms, as did type effects at these times and sites, and occipitotemporal sites also showed LPC effects after 400 ms.

#### N400

Focal spatiotemporal results (Table 2; Figure 4C) at centroparietal sites showed a category type effect from 400 to 500 ms, congruity effects from 300 to 500 ms, and congruity by type interactions from 200 to 500 ms. The N400 for faces was larger over the left hemisphere, as demonstrated by three-way interactions of type by congruity by hemisphere that were significant from 300 to 400 ms and marginal before and after [200–300 ms *F*(1, 17) = 3.05, *p* = 0.099; 400–500 ms *F* = 3.89, *p* = 0.065]. Planned simple effects tests (Table 2) from 200 to 300 ms showed no N400 effects for faces, and, instead, N3 congruity effects for objects with inverted polarity at these sites (i.e., most negative for congruous) as observed at adjacent occipitotemporal sites (Figure 3). Later, from 300 to 400 ms, congruity affected faces in the predicted direction (i.e., most negative for incongruous), as N400 congruity was significant for faces. From 400 to 500 ms, posterior LPC congruity effects began: Congruity was significant for objects and congruity by hemisphere was marginal for faces, as the effect was larger on the right (*F*[1, 17] = 4.12, *p* = 0.058). In sum, centroparietal sites showed occipitotemporal polarity inversion of N3 congruity effects for objects, N400 congruity effects for faces, and, after 400 ms, the start of posterior LPC congruity effects (Figures 2–4).

### 500–900 ms: LPC

As the earlier centroparietal focal results indicated, LPC congruity effects began around 400 ms. Posterior positivity is greater for incongruous than congruous pictures (Figures 2, 3, and 5C) and for objects than faces (Figure 7). Accordingly, omnibus results showed category type and congruity effects from 500 to 900 ms and congruity by type interactions from 500 to 700 ms (Table 1), reflecting continuing frontal type effects and frontocentral congruity effects with faces, as reported above for frontal focal results, and continuing LPC type effects until 900 ms and congruity effects until 700 ms. Focal results at centroparietal pair 19-20 confirmed the posterior distribution of the LPC, showing category type and congruity effects (Table 2); as congruity and type did not interact, congruity affected both category types. Planned simple effects tests (Table 2) showed that congruity effects continued until 700 ms for objects and were in the same direction for faces (i.e., more positive for incongruous) but did not reach significance [500–700 ms: congruity by hemisphere, *F*(1, 17) = 2.28, *p* = 0.15] perhaps due to ongoing frontal congruity effects in the opposite direction that may partly cancel out the posterior LPC effect for faces.

### P3 peak latency and amplitude

Visual inspection revealed prominent parietal P3-like peaks between 300 and 700 ms, resembling immediate perceptual repetition priming that makes the P3 earlier and larger (Bentin and McCarthy, 1994). Likewise, here, the P3 appeared to peak earlier for congruous than incongruous stimuli, resulting in a P3 that is more positive for congruous than incongruous initially and then later shows the opposite (Figures 2, 3, and 5A). Results of ANOVAs on local positive peak latency data at midline occipitoparietal site 30 confirmed that the P3 peaked earlier for congruous (426 ms) than incongruous (496 ms) stimuli [congruity, *F*(1, 17) = 11.41, *p* = 0.004], regardless of category. Peak latency captured the P3 pattern better than mean amplitude due to overlapping N400 and LPC effects. P3 mean amplitude results at site 30 showed only that the P3 was more positive for congruous than incongruous faces from 300 to 700 ms and marginally the opposite (more positive for incongruous than congruous) for objects from 400 to 500 ms; there were significant effects of congruity (300–400 ms, *F* = 20.61, *p* < 0.001) and congruity by type (300–700 ms: *F*s > 13, *p*s < 0.003) due to congruity being significant for faces (300–500 ms, *F*s > 7.45, *p*s < 0.015) and marginal for objects (400–500 ms, *F* = 3.63, *p* = 0.074).

### Mental imagery sources

Because faces and objects recruit distinct occipitotemporal areas (Hasson et al., 2003), the cortical sources of mental imagery should differ between these categories. Estimated cortical sources of each of the four difference waves were consistent with known prefrontal and posterior face (object) processing areas. MNI coordinates are reported for the maximum activated region (*x y z*) and up to four anatomically distinct sources, the Brodmann’s areas (BA) for all, and the BA for up to four secondary sources that are contiguous with the maximum; this captured all clear sources.

#### Congruity (incongruous–congruous)

For faces, results were consistent with ERP and fMRI evidence for prefrontal and temporal lobe generators during recognition and priming of faces (Henson et al., 2003). Specifically, congruity for faces from 200 to 400 ms during the N3 and N400 (and overlapping P3) reflects sources in medial prefrontal cortex, VLPFC, and superior temporal cortex: Figure 4B shows sources observed, from 200 to 300 ms, in medial frontal gyrus [BA9 at 5 45 25 and, from 300 to 400 ms in VLPFC (BA47 at 15 35 −30; BA11)] and superior temporal gyrus (STG; BA38). After 400 ms, P3 and LPC congruity reflect sources in prefrontal and middle temporal cortex: Figure 5B shows sources observed in middle temporal gyrus (400–500 ms BA21 at 70 −35 −5; 500–700 ms BA21 at 70 −35 −10) and, after 500 ms, also in medial and superior frontal gyrus (not shown; 500–600 ms, BA9/10 at 0 55 25; 600–700 ms, BA9/10 at 5 60 30).

For objects, N3 congruity effects occurred from 200 to 400 ms and so this time was of primary interest. Results were consistent with N3 and fMRI evidence for ventral object processing stream generators during categorization and priming (Henson et al., 2004; Schendan and Stern, 2008; Schendan and Maher, 2009; Schendan and Lucia, 2010). Specifically, during the N3 (Figure 4B), sources were observed, from 200 to 300 ms, in middle temporal gyrus (BA21 at 70 −35 −10) extending to inferior (BA37) and superior temporal (BA22) and fusiform (BA37) and middle occipital gyri (BA19) and, from 300 to 400 ms, at the junction of posterior fusiform and inferior occipital gyri (BA18 at 25 −90 −25 and 35 −90 −20) extending posteriorly to lingual (BA17) and anteriorly to fusiform (BA20 at −45 −25 −30; BA 37) and parahippocampal gyri (BA36). Afterward, during the later P3 peak to incongruous objects and the LPC (Figure 5B), various ventral stream sources continued, and prefrontal ones occurred initially from 400 to 500 ms: Sources were observed from (i) 400–500 ms, in VLPFC (BA47 at 20 30 −30), (ii) 400–700 ms, in fusiform (400–500 ms, BA37 at 55 −55 −25; 500–700 ms, BA20 at 55 −40 −30; BA36 at all times; BA19 at 400–500 ms), (iii) 400–900 ms, in inferior (BA20 and 37; a maximum also from 800–900 ms at BA20 at −60 −55 −20) and middle temporal gyri (BA20 until 700 ms), (iv) 600–700 ms, in parahippocampal gyrus (BA36).

#### Category type (object–face)

As expected for domain-specificity (Downing et al., 2006), objects and faces differed primarily in object and face-sensitive areas of the ventral visual pathway (Figure 6B shows only 200–400 ms as later sources remained similar). Specifically, incongruous stimuli showed sources (i) continuously until 900 ms in inferior temporal (maximum 200–300 ms: BA20 at 60 −35 −20; maximum 400–500 ms: BA37 at 60 −55 −10) and (ii) middle temporal gyri (maximum 300–400 for BA20 at 60 −45 −20), (iii) in fusiform gyrus at most times (BA36, 37: 200–500 ms), and (iv) in middle occipital gyrus from 300 to 500 ms (BA37; BA19). Likewise, congruous stimuli also showed category differences in these regions: Sources were observed (i) in middle temporal gyrus (200–300 ms maximum for BA37 at 50 −40 −15; BA20), (ii) from 200 to 300 ms in fusiform gyrus (BA36/37), (iii) at most times in inferior temporal gyrus (BA37 at −60 −65 −10; BA20: 300–500 ms), (iv) from 300 to 400 ms in middle occipital gyrus (BA19/37). In addition, congruous stimuli showed sources of category differences in (v) STG from 400 to 500 ms (BA22 at 70 −25 5; BA41/42), consistent with superior temporal face-specific processes (Puce and Perrett, 2003).

### Perception control ERPs

**Figure 10:**
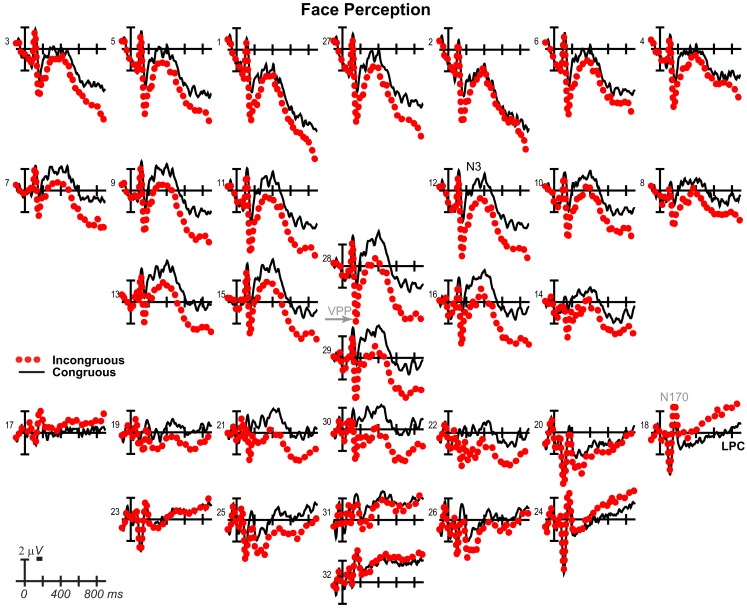
**Perception congruity effects for faces**. Grand average ERPs between −100 and 900 ms at all 32 channels shown filtered low-pass 30 Hz. LPC, late positive complex. For congruous, pictures of two different people’s faces were presented sequentially, and for incongruous, a picture of a non-face object preceded a picture of a face.

**Figure 11:**
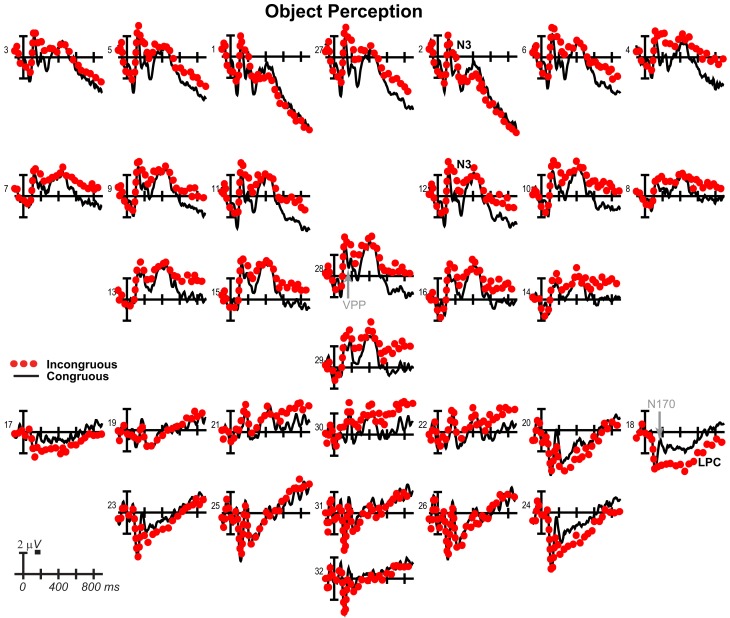
**Perception congruity effects for objects**. Grand average ERPs between −100 and 900 ms at all 32 channels shown filtered low-pass 30 Hz. LPC, late positive complex. For congruous, pictures of two different objects were presented sequentially, and for incongruous, a picture of a face preceded a picture of an object.

Results have already been reported for face ERPs before 500 ms and comparisons of the early VPP/N170 between perception and mental imagery of faces, demonstrating typical perceptual adaptation reduction of the VPP/N170 for repeated faces, as well as adaptation of later ERPs until 500 ms (Ganis and Schendan, 2008). Here, we focus on results for faces after 500 ms, results for objects, and comparisons between faces and objects. For faces (Figure 10), adaptation continued until 900 ms [i.e., more negative anteriorly and more positive at occipitotemporal sites for congruous (adapted) than incongruous], as shown by significant effects of congruity [700–899 ms *F*(1, 17) = 8.47, *p* = 0.01], congruity by electrode [500–699 ms *F*(12, 204) = 8.91, *p* = 0.001; 700–899 ms *F* = 15.88, *p* < 0.001], congruity by hemisphere [500–699 ms *F*(1, 17) = 15.63, *p* = 0.001; 700–899 ms *F* = 11.72, *p* = 0.003], and congruity by electrode by hemisphere [700–899 ms *F*(12, 204) = 2.37, *p* = 0.048]. Objects largely showed the opposite (Figure 11), resembling instead ERPs during rapid masked repetition priming that is associated with faster response times when the prime is more similar, relative to different from, the target (i.e., a positive priming benefit; Forster and Davis, 1984; Eddy et al., 2006). Such masked priming is associated with modulations of the VPP (labeled N190 in such work), frontal N3, and centroparietal N400, which are more negative for incongruous than congruous objects; an occipitotemporal N170 (labeled P190) and occipitotemporal N3 counterpart show the opposite (Eddy et al., 2006; Eddy and Holcomb, 2010). However, the present results would suggest that such positive priming benefits any category within the domain of non-face objects (i.e., between different non-face, basic, object categories) relative to the cross domain case (i.e., between faces and non-face objects), whereas all ERP masked object priming studies to date compared identical object pictures to unrelated non-face objects (Eddy et al., 2006, 2007; Eddy and Holcomb, 2009, 2010, 2011). To capture this similarity between the present object perception results and the prior masked object priming findings, the times and sites of masked priming effects were analyzed (Eddy et al., 2006; Eddy and Holcomb, 2010). Results confirmed the similarity of the present results to those in studies of masked priming: From 100 to 250 ms at frontocentral pair 11-12 for the VPP (i.e., N190), congruity was significant [*F*(1, 17) = 29.34, *p* < 0.001] and marginal at pair 23-24 for the occipitotemporal N170 (i.e., P190; *F* = 3.44, *p* = 0.081), significant from 250 to 350 ms for the N3 at frontocentral pair 11-12 (*F* = 21.84, *p* < 0.001), and 350–500 ms for the N400 at centroparietal pair 19-20 (*F* = 23.49, *p* < 0.001), but the centroparietal effect appeared to reflect only the overlapping LPC, suggesting minimal N400 modulation if any.

For completeness, the same focal spatiotemporal analyses compared perceptual congruity between types, as done for imagery, and a 140–180 ms time window was added to assess the VPP/N170, as had been done previously for faces (Ganis and Schendan, 2008). Results confirmed that perceptual congruity effects differed between categories during the VPP/N170, frontal N3, centroparietal N400, and posterior LPC. Specifically, at all times after 200 ms [all *df*s (1, 17)], frontopolar ERPs showed effects of type (type at 500–899 ms *F*s > 5.72, *p*s < 0.03; type by hemisphere at 200–899 ms *F*s > 11, *p*s < 0.005) and congruity (200–899 ms: congruity *F*s > 43, *p*s < 0.001; congruity by hemisphere *F*s > 10, *p*s < 0.006), and their interaction (type by congruity by hemisphere at 200–899 ms *F*s > 4.99, *p*s < 0.04). Frontocentral ERPs showed effects of type during the VPP and after 500 ms (140–180 and 500–899 ms *F*s > 7.96, *p*s < 0.02), effects of congruity at all times (congruity at 140–180 and 200–899 ms *F*s > 6.2, *p*s < 0.03; congruity by hemisphere at 200–299 ms *F*s > 4.91, *p*s < 0.042), and their interaction during the VPP and N3 (type by congruity by hemisphere at 140–180 and 200–299 ms *F*s > 6.31, *p*s < 0.01). Occipitotemporal ERPs showed effects of type on the N170 and LPC (type at 140–180 and 400–499 ms *F*s > 4.65, *p*s < 0.05; type by hemisphere 200–299 ms *F*s > 12, *p*s < 0.003), and only the N170 showed effects of congruity (140–180 ms, *F* = 15.81, *p* = 0.001) and the interaction (type by congruity at 140–180 ms, *F* = 16.80, *p* = 0.001). Centroparietal sites showed effects of type (type by hemisphere at 200–499 ms, *F*s > 6.02, *p*s < 0.03), congruity (400–499 ms *F*s > 6.77, *p*s < 0.002), and interactions of type by congruity (200–499 ms *F*s > 7.21, *p*s < 0.02), but reflected N400 adaptation for faces and instead overlapping LPC priming for objects. After 300 ms, the LPC at posterior pair 25-26 showed effects of type (300–499 ms *F*s > 8.31, *p*s < 0.02) and the interactions (type by congruity at 500–899 ms, *F*s > 4.94, *p*s < 0.05; type by congruity by hemisphere at 300–399, *F*s > 6.56, *p*s < 0.03).

### Performance

As stimulus timing is critical to interpreting the results, time to report mental image generation (mental imagery experiment) or identification (perception experiment) is reported in more detail than before (Ganis and Schendan, 2008). Results of an ANOVA with experiment (imagery, perception), congruity, and category factors demonstrated that timing differences cannot explain category and congruity effects. Mental imagery was slower than perception [*F*(1, 17) = 267.30, *p* < 0.001], as subjects took a long time, 3,889 ms, from the onset of the word cue to report generation of the mental image, and only 1,070 ms from the onset of the first picture during perception to report identification of the face (categorization of the object); note, with the 200 ms ISI included, these times corresponded to an average, stimulus onset asynchrony (SOA) of 4,089 ms for imagery (*SD* = 792 ms; range 2,548–5,252 ms) and 1,270 ms for perception (*SD* = 346 ms; range 754–2,113 ms). Critically, these SOA times were indistinguishable between category and congruity conditions (*F*s < 1, *p*s > 0.8), and, no interaction was significant (*F*s < 2.1, *p*s > 0.17). Specifically, for mental imagery, SOAs were similar between categories and between congruous (faces 4,071 ms; objects 4,107 ms) and incongruous conditions (faces 4,081 ms; objects 4,096 ms). Likewise, for perception, SOAs were similar between categories and between congruous (faces 1,311 ms; objects 1,224 ms) and incongruous conditions (faces 1,247 ms; objects 1,298 ms).

## Discussion

The findings reveal the cortical dynamics of ongoing top-down processes of mental imagery of visual shape during later knowledge, meaning, and decision processing of a perceived picture. Overall, ongoing mental imagery facilitates categorical perception during the early VPP/N170, as reported previously (Ganis and Schendan, 2008), and higher cognitive processes during later ERPs, as reported here. Specifically, the frontal N3(00) complex, which indexes object and face cognition, knowledge, and category decisions from 200 to 400 ms and the centroparietal linguistic N400 index of semantic memory from 300 to 500 ms are more negative for incongruous than congruous stimuli. Notably, N3 and N400 effects dissociate from each other not only temporally (i.e., earlier for N3) and spatially (i.e., more frontal for N3) but also in how congruity effects differ between categories. N3 effects are frontopolar for objects and frontocentral for faces and associated with different brain sources between categories. While the N400 congruity effect is small but clear for faces, it is smaller, and, indeed, minimal and not clearly evident for objects. In addition, the parietal P3 peaks earlier for congruous (∼400 ms) than incongruous (∼500 ms) stimuli. After 400 ms, the posterior LPC shows the opposite congruity effect from the N3 and N400, being instead more positive for incongruous than congruous stimuli until 700 ms. This pattern of effects resembles a combination of ERP effects of semantic congruity (on N3, N400, and LPC) and immediate repetition priming (on N3, N400, and P3). In contrast, perception shows a different pattern of congruity effects, as predicted due to the bottom-up contributions to perception but not imagery. Further, the pattern differs between categories: Faces show adaptation; objects show rapid priming effects in the opposite direction. Altogether, these findings indicate that top-down processes of mental imagery can induce a powerful imagistic mental representation of visual shape that mimics top-down processes recruited also for picture perception and facilitates knowledge, meaning, and categorization processes.

### Mental imagery

Next we consider the evidence that these mental imagery effects are related to semantic congruity and immediate repetition priming processes and the implications of this for how mental simulation can ground cognition in shape processing. First, it is important to be reminded of key, unique aspects of the present methods (Ganis and Schendan, 2008). (a) People had extensive training generating mental images of each picture of a real person or object, a standard procedure used in validated mental imagery tasks. (b) The name (i.e., written word) for the person or object cued subjects to visualize mentally each associated trained picture. (c) A picture probed ongoing mental imagery of faces and objects, and ERPs were recorded to this picture, which either was the picture subjects were cued to visualize mentally or another picture from the opposite category. Further, two categories (faces and objects) that are supported by different visual processing areas (Hasson et al., 2003; Downing et al., 2006; Tsao et al., 2006) were used to manipulate congruity, and the opposite category was assigned to the incongruous condition. Consequently, congruity effects reflect large differences in shape processing. This is because congruent face imagery (identical face) maximally affects face processes, while incongruent imagery (object) minimally affects face processes, and analogously for object imagery. (d) The delay between the name cue and target picture was relatively long (SOA 4,089 ms, on average). (e) No task was performed on the target picture to minimize decision and response related ERPs that can complicate interpretation of the waveform, thereby defining knowledge and semantic memory processes as clearly as possible. While a limitation of this study is that the target picture was not followed by a task that assessed the mental imagery, evidence that subjects did as instructed is that they took much longer (2,819 ms) to visualize the named picture in the mental imagery experiment than to identify the perceived face (object) picture in the perception control experiment.

#### Mental imagery produces semantic congruity effects and facilitates cross modal priming

The most important finding here is that mental imagery produces ERP effects that resemble N3, N400, and LPC effects observed in studies of short latency, cross modal priming, semantic congruity, and semantic priming phenomena. No prior behavioral or ERP study on these phenomena or mental imagery would have predicted this finding, as mentioned in the introduction. One reason is that the timing for mental imagery here is well beyond that for automatic spreading activation associated with semantic priming, which is thought to underlie semantic congruity effects and to reveal semantic memory processes (Kutas and Federmeier, 2011). Consider that, in the typical semantic priming task, two related words are presented sequentially with a brief delay (usually under 1 s): A target word (e.g., “doctor”) is preceded by a prime word that is different and either semantically related (“nurse”) or unrelated (“truck”) to the target. Response times are faster to targets preceded by primes that are semantically related (congruous) than unrelated (incongruous). Findings with SOAs between prime and target of under 500 ms reflect automatic spreading activation in the semantic network, whereas SOAs between 500 and 1000 ms or so reflect controlled semantic analysis (Rossell et al., 2003; O’Hare et al., 2008). Thus for the timing in our study (for both imagery and perception), the results can reflect only controlled semantic processing. In contrast, most semantic priming work studied automatic spreading activation using short SOAs under 500 ms and so evidence of behavioral and ERP priming with longer delays is scarce and more so for the much longer delays here.

A second reason these findings are novel is that the procedure here of having a word (the name) precede a picture is used in cross modal priming studies. Critically, for word-picture priming, behavioral evidence has been mixed and, if such cross modal priming is found, it occurs mainly with much shorter delays than used here. Such studies typically use an SOA of about 1 s (Bajo, 1988) and often much less (Carr et al., 1982) but also often mask the prime. At such short SOAs, word-picture priming can be comparable (Bajo, 1988) or much less than within modality (e.g., picture to picture; Carr et al., 1982). At slightly longer SOAs of less than about 2 s, priming can be absent (Biggs and Marmurek, 1990). Like behavioral effects, ERP effects of cross modal priming (on the N3, N400, and P3/LPC) have been found most consistently in studies using SOAs briefer than 500 ms or unmasked primes composed of multiple words in sentences or noun phrases (Ganis et al., 1996; Federmeier and Kutas, 2001, 2002; Stanfield and Zwaan, 2001; Zwaan et al., 2002; Hirschfeld et al., 2012). Cross modal priming from a single word to a picture is more variable but has been found at SOAs ranging from 120 to 700 ms on the N400 (and perhaps P3/LPC) between 350 and 550 ms when the prime is unmasked (Auchterlonie et al., 2002; Johnson and Olshausen, 2003, 2005; Dobel et al., 2010; Kiefer et al., 2011) and masked (Blackford et al., 2012). In contrast, N3 cross modal priming has been found only in studies that (a) mask the word prime, use the shortest SOAs (120 ms or less), and overt naming, or (b) use long SOAs of about 1–2 s and name verification (Johnson and Olshausen, 2003, 2005). Notably, for priming from a word to a picture, visually impoverishing the objects (by occlusion or fragmentation) yields a more frontopolar distribution of congruity effects. This scalp distribution is consistent with the frontopolar N3 in studies of object cognition and priming with non-canonical views, fragmented real objects, and pseudo objects (Holcomb and McPherson, 1994; Schendan et al., 1998; McPherson and Holcomb, 1999; Schendan and Kutas, 2002, 2003, 2007; Schendan and Lucia, 2009; Schendan and Maher, 2009), which recruit top-down processes more (Michelon et al., 2003; Ganis et al., 2007), and as found here for mental imagery of objects. In sum, behavioral and ERP (N3, N400, P3/LPC) effects of cross modal priming can occur at shorter SOAs, with multiple words as the prime, and during naming tasks, but, crucially, none of these procedures apply to the mental imagery task used here.

In contrast, for long SOAs well beyond about 1 s and more like mental imagery here, behavioral cross modal priming from a single word to a picture has not been found (Morton, 1979; Warren and Morton, 1982) or is much smaller than that within modality (Carr et al., 1982). The ineffectiveness of word primes for picture targets at long delays, however, can be overcome, by (a) varying prime modality only between- (i.e., not within-) subjects, (b) blocking prime modality (Brown et al., 1991), or (c), critically here, instructing subjects to use mental imagery. A word that is used to cue mental imagery during a study session primes later performance with the picture at a long delay (minutes) on an implicit memory test with objects (McDermott and Roediger, 1994) or famous faces (Cabeza et al., 1997) and can do so as well or better than a perceived picture (Michelon and Koenig, 2002; Michelon and Zacks, 2003). However, it is important to note that these priming studies do not use the picture target at test to reveal ongoing mental imagery sustained within working memory, as herein, but rather its long-term consequences for a memory test much later (i.e., beyond the time limits for working memory). Notably, prior ERP studies, in which a single word could prime a picture target at a long delay, have not used blocking or mental imagery procedures, and, accordingly, no behavioral or ERP priming effects were found. Although episodic recognition does show effects (Kazmerski and Friedman, 1997; Spironelli et al., 2011), these do not apply here because we assess ongoing mental imagery, not the consequences for later episodic recollection. Further, even if N400 effects are found at such long lags, they likely reflect morphological (linguistic) representations, not semantic or phonological representations which do not seem to survive lags beyond SOAs of 300 ms (Feldman, 2000; Koester and Schiller, 2008). This would suggest that N400 effects for mental imagery here reflect linguistic, not semantic, memory representations (Kousta et al., 2011), but future work needs to assess this.

#### Mental imagery primes perception like immediate picture repetition does

Altogether, these direct neurophysiological findings are consistent with behavioral evidence that mental imagery facilitates object categorization via priming mechanisms (Peterson and Graham, 1974). Most striking is the finding that the N3, N400, and P3 congruity effects mimic ERP immediate repetition priming, providing direct neurophysiological evidence that mental imagery can affect neural processing like actual perception of a picture can. When the exact same image repeats immediately with no intervening stimuli, ERPs after 300 ms become earlier and larger for objects (Nielsen-Bohlman and Knight, 1994; Zhang et al., 1995), faces, and words (Bentin and McCarthy, 1994; Schendan et al., 1997). The P3 is more positive and peaks earlier (∼400 ms) and the following N400 is more positive for repeated (akin to congruent) than unrepeated (akin to incongruent) faces and objects, and a later P3 or LPC, peaking around 500 ms, is instead more positive for unrepeated than repeated pictures. These ERP effects of perceptual immediate repetition priming have been observed at relatively short SOAs of 1200–3500 ms, which is much longer than the 500 ms SOA necessary to observe automatic spreading activation in semantic priming and slightly longer than the about 1 s SOA for cross modal priming (without special conditions like mental imagery). For objects, most studies could not or did not assess frontal ERPs, but one study also shows the frontal N3 is more positive for repeated than unrepeated items (SOA 2400 ms; Henson et al., 2004). This study also reported VPP/N170 repetition adaptation for objects (i.e., smaller for repeated), but this adaptation direction is the opposite of the later repetition priming effects in that study and of the mental imagery effects here, and no other immediate repetition study found effects before 200 ms (Nielsen-Bohlman and Knight, 1994; Zhang et al., 1995). Altogether, these findings indicate that mental imagery mimics the pattern of immediate repetition priming of perceived pictures on the N3, N400, P3, and LPC. However, mental imagery also enhances the VPP/N170, unlike immediate repetition priming, which typically has little or no effect on early ERPs.

The similarity between mental imagery effects and ERP immediate repetition priming is consistent with the role of working memory in both. Immediate repetition priming is due to working memory for the first image that is sustained across the brief delay until the second image appears (Bentin and McCarthy, 1994). This working memory facilitates categorization of the repeated percept with minimal or no reactivation of semantic memory, minimizing the N400. Likewise, the priming effect on the frontal N3 could indicate that visual knowledge and cognitive decision processes for objects and faces are also largely bypassed. Immediate repetition effects on the P3 reflect modality-specific (i.e., visual) working memory that speeds the category decision (Bentin and McCarthy, 1994; Nielsen-Bohlman and Knight, 1994; Zhang et al., 1995). Consistent with maintaining modal visual information in working memory, P3 facilitation is not associated with semantic priming, as reviewed above. Further, immediate repetition effects on the P3 are likely also due to having subjects perform a task on the pictures, which was often episodic recognition, as such task requirements maximize P3 and other late posterior positivities (Dien et al., 2004). Mental imagery had no task requirements for the target picture, but the practice session required subjects to assess how well their mental image matched the picture; thus subjects likely continued to do so incidentally during the mental imagery test, resulting in P3 facilitation despite no overt task. Thus, mental imagery can simulate the top-down cortical dynamics that are produced by an actual perceived picture, and the episodic memories encoded during training and practice contain visual details sufficient to enable mental imagery representations to operate like an actual perceived picture (as in immediate repetition priming). This provides strong and direct neurophysiological support for the pictorial theory of mental imagery and implicates these strong pictorial representations in episodic memory of personally experienced, autobiographical information that depends upon the mediotemporal lobe (Ganis and Schendan, 2011). This finding also constitutes evidence for the visual detail achievable by the episodic memory system. Such evidence will be crucial for developing theories of mental simulation for episodic memory (Schacter et al., 2008).

#### Reflexive top-down processes for mental imagery support automatic mental simulation

By using faces and objects, which have partially non-overlapping visual processing pathways, these mental imagery findings define the largest possible set of top-down mechanisms that support mental simulation of face (object) shape, including non-conscious automatic simulation. Mental simulation has been proposed to operate via a pattern completion process that re-enacts modal processing that had occurred during learning when later retrieving the memory (Barsalou, 2009). We proposed that, at the level of brain mechanisms, the top-down feedback mechanisms that support automatic simulation are a subset of those that support mental imagery (Ganis and Schendan, 2011). Specifically, automatic simulations unfold via reflexive top-down signals from higher to lower level areas along modal information processing pathways, such as the ventral stream: Perceiving a stimulus triggers these processes reflexively (Ganis and Kosslyn, 2007). Through such distributed multi-regional activity, seeing an object or reading its name (e.g., “dog”), for example, re-enacts associated modal features that were stored during earlier learning experiences (e.g., its shape, color, motion, actions with it), thereby constructing cognition, memory, and meaning. This is consistent with the MUSI account that proposes top-down feedback processes after 200 ms have the greatest role in visual cognition, constructing knowledge, meaning, memory, and decisions (Schendan and Kutas, 2007; Schendan and Maher, 2009; Schendan and Lucia, 2010). These same processes are triggered by strategic top-down signals from the prefrontal cortex during mental imagery (Ganis and Kosslyn, 2007) and so mental imagery time courses like those here can define when and how mental simulation grounds cognition. Previously, semantic priming has revealed the most about automatic mental simulation and its brain basis, especially with words (e.g., Marinkovic et al., 2003; Rossell et al., 2003; Kutas and Federmeier, 2011). This is because automatic spreading activation across a semantic memory network, which explains such priming, is thought to operate via the same automatic and reflexive, top-down processes that have been implicated in automatic simulation (e.g., Collins and Loftus, 1975; Franklin et al., 2007; Kutas and Federmeier, 2011). The similarity between the results here using a validated mental imagery task and ERP findings related to semantic congruity and immediate repetition priming supports this conclusion.

### Perceived picture identification adapts faces but primes objects

Overall, perception control results confirm that common top-down processes underlie similarities between imagery and perception, while bottom-up processes for perception (but not imagery) underlie their differences (Ganis and Schendan, 2008). The time precision of ERPs offers advantages over fMRI and behavior for characterizing such similarities and differences between perception and imagery. Specifically, perception results dissociate between categories, consistent with the domain-specificity of object and face processing (Downing et al., 2006): Perceptual repetition adapts processing of perceived faces from categorical perception onward, as predicted, but instead unexpectedly primes processing of objects during categorical perception, visual knowledge processing, and strategic semantic analysis. Critically, identification time for the first picture is similar for objects and faces and so cannot explain differences in congruity effects. In fact, the timing was chosen to replicate classic face adaptation effects on the VPP/N170(Jeffreys, 1996) obtained with an 1,100 ms SOA, 800 ms duration, and 300 ms ISI (i.e., like the 1,070 ms identification RT and 200 ms ISI here), as was achieved (Ganis and Schendan, 2008). To understand the perception control findings, it is necessary to highlight that both prime and target were always different pictures, even in the congruous condition. Thus, perception results show how perceiving a picture of a face (object) is affected by having previously identified a perceived picture of a different face (object), compared to having previously identified the opposite category picture [i.e., of an object (face)].

For faces, seeing two different people sequentially adapts the ERPs, thereby producing the opposite congruity effect from that for mental imagery. For congruous relative to incongruous perception, the VPP is less positive (and N170 less negative), as reported previously (Ganis and Schendan, 2008), the N3 and N400 are more negative, and the LPC is more positive. Altogether this finding and the mental imagery finding indicate that early congruity effects on the VPP/N170 extend to later ERPs. The direction of these effects suggests their interpretation. Consider that all these adaptation effects go in the opposite direction compared to the facilitation effects found for mental imagery: Adaptation reduces the VPP/N170 but enhances later ERPs. Such enhancements of later ERPs are thought to reflect greater (not less) recruitment of the underlying processes (Schendan and Kutas, 2003). Hence, early adaptation of categorical perception during the VPP/N170 impedes later cognitive processing (i.e., due to failure of an earlier critical process), thereby recruiting additional top-down processing resources to accomplish these later cognitive functions (Kosslyn et al., 1994; Ganis et al., 2007).

The direction of the object perception findings tells a different story, which further supports a facilitation interpretation of the mental imagery findings because the direction of the effect is the same as for imagery. Specifically, under rapid, immediate serial presentation, the perception of an object picture primes (facilitates) a subset of the ERPs to a target object picture that mental imagery also primes. For congruous relative to incongruous conditions, the VPP is more positive (and N170 more negative), the N3 less negative, and LPC less positive, whereas the N400 shows minimal or no priming. Unlike imagery, though, perceptual repetition shows no P3 modulation, but this is presumably due to no overt or implied task on the perceived target picture. Surprisingly, therefore, priming of whatever processes are shared among a set of real objects from different basic categories can facilitate processing of each other (i.e., congruous perception), in contrast to the cross domain case of perceiving a face and then a non-face object (i.e., incongruous perception). The resemblance between these perception (and the mental imagery) results and those for certain kinds of rapid perceptual and semantic priming supports a facilitation interpretation. After all, the N3, N400, and LPC effects of object perception congruity resemble a subset of effects for mental imagery and immediate repetition and semantic priming. Moreover, the waveforms resemble those associated with priming under the most rapid, serial presentation of pictures (SOA < 500 ms) when the prime is either not masked and semantically related pictures repeat (Holcomb and McPherson, 1994; McPherson and Holcomb, 1999; Kiefer et al., 2011) or masked and identical pictures repeat (Eddy and Holcomb, 2010). In masked repetition priming, the VPP (labeled N190 in these studies), N3, and N400 are more negative [and occipitotemporal N170 (labeled P190) and N3 counterpart are more positive] for unrelated than repeated (identical) pictures of objects (Eddy et al., 2006; Eddy and Holcomb, 2010). The waveform similarity between object perception here and masked priming must be due to the very short 200 ms ISI used here for perception, causing the ERPs to the prime and target to overlap temporally, as they do in the masked priming work, which uses short ISIs of 100 ms or less. The short ISI is probably also responsible for some of the effects resembling effects for rapid repetition and masked semantic priming (with ISIs of 200 ms or less) more than for longer lag, immediate repetition priming (with SOAs of 1200–3500 ms). In turn, the longer SOA here may explain why the later perceptual congruity effects also resemble some longer lag, immediate repetition priming effects on the N3 and LPC at SOAs of about 1 to 1.5 s, which show the same pattern of congruity effects on the N3, N400, and a posterior P3/LPC (Barrett and Rugg, 1990; Holcomb and McPherson, 1994; McPherson and Holcomb, 1999; Bach et al., 2009). Thus, for objects (but not faces) perception-driven priming and mental imagery congruity effects differ quantitatively and qualitatively but nonetheless all follow a direction that indicates priming facilitation. The similarity between the perception and imagery ERP congruity effects with objects further bolsters the idea that mental imagery mimics perception: Mental imagery of objects, primes (facilitates) object processing like repeating a perceived picture.

### Caveats

Future work will need to investigate why, despite identical timing, perception of faces and objects produce opposite congruity (repetition) effects on the ERPs to the probe picture. N170 rapid adaptation evidence (Nemrodov and Itier, 2012) suggests that faces are stronger adaptors than some non-face categories. Face (and car) primes reduce (i.e., adapt) the N170 more than chair and house primes for all categories of test objects (i.e., faces, cars, chairs, houses). Prime category and ISIs are the key factors determining how much, if at all, the prime adapts the test stimulus; after all, the ISIs of 232–268 ms and test stimulus duration of 200 ms resemble the present timing, but the faces were of unknown people, whereas here they were famous, and the prime was much briefer (i.e., 200 ms) than here, suggesting neither knowledge nor prime duration can explain the findings. The reason that the category of the prime matters is unclear but generally consistent with transfer appropriate processing and encoding specificity accounts of memory (Tulving and Thomson, 1973; Morris et al., 1977). Other proposals include interference from ongoing late processing of the adaptor due to the short ISI and neural fatigue that is not category specific but selectively tuned to adaptor properties (Nemrodov and Itier, 2012). Regardless, faces can adapt more than other object categories. The present results suggest an additional twist: Non-face objects can prime better than faces. One may speculate that, here (and in related work), face perception shows a substantial adaptation pattern because faces (congruent) adapt better than other objects (incongruent), while objects prime better, and object perception shows a substantial priming pattern because objects (congruent) prime better than faces, while faces (incongruent) adapt better; both adaptation and priming influences could affect any result, making congruity effects larger than either influence alone. The relative strengths of adaptation and priming explain the pattern observed, which will be important for future research to tease apart.

This caveat also speaks to an alternative hypothesis to explain why both perception and mental imagery prime, but perception also adapts a stimulus: Perception adapts not only face (object) processing but also adapts visual sensory processing, and the latter results in reduced sensitivity in lower-level areas, including occipital cortex (Wilson and Humanski, 1993; Anderson and Wilson, 2005; Loffler et al., 2005). This is another way of stating that both perception and mental imagery can recruit top-down processes, while perception is also driven by bottom-up processes (Ganis and Schendan, 2008) but adds the idea that perception also adapts lower-level occipital areas. After all, both perceptual priming and adaptation effects on perception are well-documented (Grill-Spector et al., 2006), and top-down processes from the frontal lobe, which support mental imagery, have been implicated in perceptual repetition priming (Schacter et al., 2007). Consider that different faces share more lower-level features, such as spatial frequency spectra, and so have less interstimulus variance than different objects (Costen et al., 1996; Thierry et al., 2007; Ganis et al., 2012). Hence, different faces can mutually adapt not only face processing but also lower-level feature processing more than different objects. For example, the N170 face-specificity effect overlaps spatiotemporally with an N1(00) component (i.e., N170 face-specificity modulates the N1), which reflects widespread bottom-up and reflexive feedback processing along the visual pathways from lower to higher level areas (e.g., Vogel and Luck, 2000; Bullier, 2001). Adaptation of both the face-specific and the low-level visual processes results in a large decrease, as both N170 face and N1 visual components are affected. In contrast, mental imagery of face-specific processes increases N170 amplitude, but this is a smaller change (than the decrease for adaptation) because only the N170 face component is affected. This explanation needs to be considered to resolve whether perception and mental imagery share properties (Freyd and Finke, 1984) or not (Craver-Lemley and Reeves, 1992).

Finally, given the novelty of this mental imagery probe paradigm (Ganis and Schendan, 2008), several issues remain to be resolved. For example, future work will need to evaluate how individual differences in mental imagery and other abilities (Kozhevnikov et al., 2005) affect mental imagery processes, as well as how congruity effects differ as a function of the vividness of the perceived stimulus and subjective vividness of mental imagery (Herholz et al., 2012). Also, mental imagery will need to be compared directly with cross modal priming from a word to a picture and potential processing differences (e.g., depth of semantic network activation) will need to be addressed.

## Conclusion

The ERP results described in this report define the neurophysiological characteristics and time course of top-down processes for mental imagery of the visual shape of faces and objects that can ground cognition in these modal processes. These findings provide striking direct neural evidence that top-down feedback processes of mental imagery sustain an imagistic representation that mimics perception well enough to prime subsequent perception and cognition like an actual picture. By manipulating congruity by switching between face and object categories, which involve different modal processes along the ventral visual stream, the ERPs reveal the largest set of top-down processes for mental imagery of these shape categories. The subset of these mental imagery processes that correspond to the reflexive top-down inputs from higher to lower level areas along the posterior ventral face (object) processing pathway also constitute the automatic mental simulation processes that can ground cognition of faces (objects). The ERP congruity effects here therefore provide direct neurophysiological markers for these visual shape mental simulations that can be used to determine precisely when, how, and how much these cortical mental simulation mechanisms ground cognition. Together, the robust frontal N3 and minimal centroparietal N400 mental imagery congruity findings confirm the visual imagistic (non-linguistic) nature of shape mental imagery, and further implicate the N3 as an index of visual knowledge (Schendan and Maher, 2009) and the N400 as an index of linguistic knowledge. This is consistent with grounded cognition distinctions between non-linguistic (“experiential” modal sensorimotor and mental state information) and linguistic systems (word-related associations) for semantic memory (Kousta et al., 2011; Paivio and Sadoski, 2011). Thus, future work on the cortical dynamics of the contribution of mental simulation of visual shape to semantic memory should focus on the frontal N3. Further, finding both mid-latency (N3, N400) and LPC congruity effects suggests that, to ground cognition in modal processing, two types of mental simulation can operate at two distinct times. Automatic simulations of visual shape (due to reflexive top-down processes) and linguistic processes operate between 200 and 500 ms during the N3 and N400, respectively, and effortful simulation (due to strategic top-down processes) operates between 400 and 700 ms during the LPC. Altogether, these markers, and others defined using the methods developed here, can be used to characterize and probe these mental simulation processes in future research on grounded cognition theory, especially for discovering the neural mechanisms of how mental simulation works.

In addition, for both imagery and perception, functional dissociations and spatial distribution differences between faces and objects provide further evidence for domain-specificity and the modality-specific processing posited for grounded cognition. In contrast to mental imagery, perception of a face (instead of an object) adapts categorical perception, which consequently impairs later processing of a different face, whereas perception of an object (instead of a face) primes categorical perception, activation of visual knowledge, and later categorization of a different object. The differences between mental imagery and perception are consistent with the strategic top-down processes required to construct and maintain mental imagery versus the bottom-up input required for perception, whereas the similarities are consistent with the common, automatic, top-down, modal processing supporting both, and further support the imagistic nature of mental imagery.

## Author Note

Preparation of this manuscript was supported by the University of Plymouth and funded by an International Research and Collaboration grant to Haline E. Schendan. The authors are grateful for the assistance of Roderick Elias, B. S., Dr. Stephen M. Maher, and Lisa C. Lucia, M.Sc. for recording the EEG data.

## Conflict of Interest Statement

The authors declare that the research was conducted in the absence of any commercial or financial relationships that could be construed as a potential conflict of interest.
